# A Method to Produce and Purify Full-Length Recombinant Alpha Dystroglycan: Analysis of N- and O-Linked Monosaccharide Composition in CHO Cells with or without LARGE Overexpression

**DOI:** 10.1371/currents.md.3756b4a389974dff21c0cf13508d3f7b

**Published:** 2013-01-02

**Authors:** Jung Hae Yoon, Rui Xu, Paul Martin

**Affiliations:** Center for Gene Therapy, The Research Institute at Nationwide Children’s Hospital; Center for Gene Therapy, The Research Institute at Nationwide Children’s Hospital; Associate Professor, Department of Pediatrics and Children's Research Institute, Ohio State University

## Abstract

α dystroglycan (αDG) is part of the dystrophin-associated glycoprotein (DAG) complex, a series of cytoskeletal, transmembrane, and membrane-associated proteins that serve to link the extracellular matrix (ECM) surrounding individual skeletal myofibers to the intracellular F-actin cytoskeleton. Glycosylation and ECM protein binding to αDG are regulated by a number of genes that, when defective, give rise to congenital or limb-girdle forms of muscular dystrophy termed dystroglycanopathies. One such dystroglycanopathy gene is LARGE. Here, we describe a method to produce and purify full-length, furin-resistant, recombinant αDG from CHO cells and CHO cells overexpressing LARGE (CHO-LARGE). In addition, we analyze the O- and N-linked monosaccharide composition of such proteins. αDG purified from CHO-LARGE cells had increased molar content of xylose and fucose relative to CHO, while no significant changes were found in N-linked monosaccharides. Glucuronic acid could not be quantified by the methods used. These studies describe a method to produce and purify the milligram amounts of αDG needed for certain biochemical methods, including monosaccharide analysis. Key words: Dystroglycan, muscular dystrophy, xylose, fucose, laminin, LARGE Correspondence: Paul.Martin@nationwidechildrens.org

## Introduction

Dystroglycan serves an essential role in preserving muscle membrane integrity by mediating binding of extracellular matrix (ECM) proteins present in the basal lamina to the F-actin cytoskeleton^1^ ^2^ ^3^. Dystroglycan is comprised of two protein subunits: alpha dystroglycan (αDG), an extracellular membrane-associated protein, and beta dystroglycan (βDG), a transmembrane protein^4^. αDG and βDG are created by post-translational cleavage from a single polypeptide precursor encoded by the dystroglycan (*Dag1*) gene^4^ ^5^. αDG is further cleaved such that the N-terminal third of the protein is removed by furin^6^, while βDG can also be cleaved by matrix metalloproteinases^2^ ^7^. Once made, αDG binds to βDG in a very tight, but non-covalent, complex^4^. αDG serves as a major binding protein for ECM proteins including laminins, agrin, and perlecan, as well as for infectious agents including viruses and bacteria, while βDG, through its cytoplasmic domain, links this complex to F-actin-binding proteins, including dystrophin^2^. Binding of many ECM proteins and infectious agents to αDG requires the proper O-linked glycosylation of αDG, which has been shown to contain core 1 O-glycans (Galβ1,3GalNAcα-O) and O-mannosyl-linked glycans (Neu5Ac or Neu5Gcα2,3Galβ1,4GlcNAcβ1,2Manα-O) in tissues and on recombinant proteins^8^^-^ 12.

Mutations in at least six genes lead to aberrant glycosylation of αDG in its mucin-rich region with O-mannosyl-linked tetrasaccharides of the type Neu5Ac/Neu5Gcα2,3Galβ1,4GlcNAcβ1,2Manα-O^13^^-^ 15. In such disorders, termed dystroglycanopathies, loss of ECM binding correlates with loss of binding to glycan-dependent α dystroglycan monoclonal antibodies such as IIH6^16^. In almost all such cases, αDG and βDG are still expressed at the sarcolemmal membrane of the skeletal myofiber, but αDG lacks important glycan structures needed for proper ECM binding, yielding muscular dystrophy which can be associated with brain and ocular malformations as well as cardiomyopathy^17^ ^18^. One such dystroglycanopathy gene is Large^myd ^or LARGE^19^, which has been recently described both as being required for synthesis of phosphate moieties on O-linked mannose glycans on αDG 20 and also as a tandem α1,3glucuronic acid (GlcA) and β1,3xylose (Xyl) glycosyltransferase^21^. LARGE may also have a therapeutic role in dystroglycanopathies, as its overexpression can stimulate increased glycosylation of αDG even in disorders where other genes in the pathway are mutated^22^. More recent work in transgenic mice also suggests that LARGE increases the apparent molecular weight of αDG, but that this may result in reduced muscle specific force^23^. The N-terminal domain of αDG, which is normally cleaved off by furin^6^, interacts with LARGE 24, and at least one mutation in the N-terminal domain of αDG gives rise to Limb Girdle Muscular Dystrophy where LARGE-αDG interactions are reduced along with αDG glycosylation^25^. In addition, one recent report suggests that several amino acids just C-terminal to the furin cleavage on αDG site bear the O-linked glycans required for laminin binding^26^. To better understand the function of LARGE, we have analyzed the monosaccharide composition of full-length (furin-resistant) recombinant αDG produced from CHO cells and from CHO cells overexpressing LARGE (CHO-LARGE).

## Results

In order to be able to perform monosaccharide analysis, we wanted to develop a method to isolate and purify full-length recombinant a dystroglycan (αDG) in milligram amounts from transfected mammalian cell supernatant. We chose CHO cells for this purpose because many glycosylation mutants have been engineered with these cells^27^. There were two major obstacles to this approach. The first was that the N-terminal third of αDG polypeptide is almost stoichiometrically cleaved in skeletal muscle and many non-muscle cells by furin, a Golgi protease^6^. Thus, the N-terminal third of the protein is normally missing from αDG after purification from cells or tissues. Second, αDG binds tightly, though non-covalently, to β dystroglycan (βDG) in the muscle membrane^4^. Thus, when the dystroglycan gene, which encodes both αDG and βDG^5^, is overexpressed in cells, a heterodimeric α/βDG protein complex is formed that co-localizes to the plasma membrane, allowing little αDG secretion.

To surmount these obstacles, we first synthesized a cDNA encoding full-length αDG with a stop codon at the end of the αDG coding sequence^28^. This allowed αDG overexpression in the absence of βDG. Because we wanted to enrich for full-length protein, we also placed a FLAG epitope tag at the N-terminus of the cDNA after the signal peptide. When this cDNA was transfected into CHO cells made to overexpress LARGE (CHO-LARGE), the recombinant secreted protein was cleaved, likely by furin, such that little to no full-length protein could be identified (Fig. 1). Addition of a cell-permeant furin inhibitor to αDG-transfected-CHO-LARGE cells, however, led to expression of highly glycosylated (ca. 120-250kDa), FLAG-tagged, secreted protein (Fig. 1). To identify secreted αDG, transfected cell supernatant was purified with anti-FLAG (M2) agarose. The ability to produce full-length αDG was specific for furin inhibition, as addition of a plasmin inhibitor had no such effect (Fig. 1). Transfection of αDG into C2C12 cells, which were then fused into myotubes, also allowed for secretion of full-length glycosylated recombinant αDG protein, purified from transfected cell supernatant using anti-FLAG agarose, in several experiments (ca. 160-180kDa), but again this only occurred in the presence of a furin inhibitor (Fig. 1). Addition of the furin inhibitor to C2C12 myotube cultures, by contrast, caused no major change in β dystroglycan expression (Fig. 1). Thus, in both muscle cells and in CHO-LARGE cells, a full-length FLAG-tagged αDG protein could be secreted in relatively large amounts as long as furin was inhibited. We chose to utilize a recombinant protein with the FLAG tag at the N-terminus of the protein to enrich for recombinant full-length protein using anti-FLAG antibodies. Therefore, we did not make or utilize a recombinant protein with a FLAG tag placed at the C-terminus of the protein.

**Inhibition of furin cleavage allows secretion of full-length recombinant alpha dystroglycan (αDG) from CHO-LARGE and C2C12 cells.**
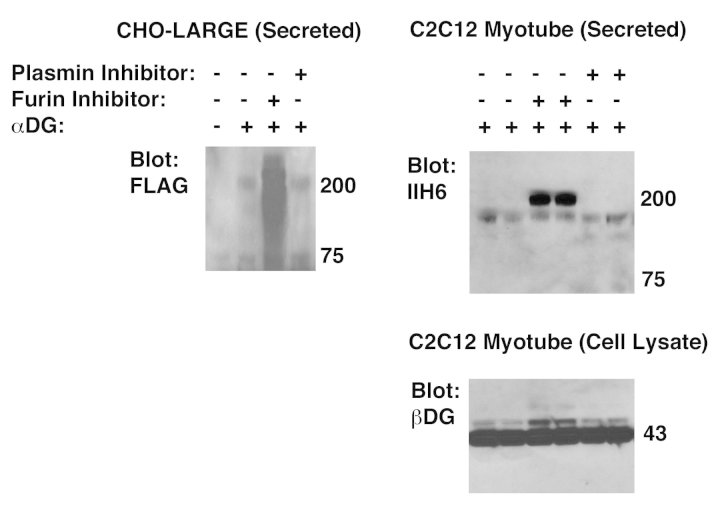
A plasmid encoding a full-length cDNA for α dystroglycan (αDG) with a FLAG epitope tag at the N-terminus was transfected into CHO-LARGE cells or C2C12 cells (which were fused into myotubes after selection of transfectants). Cells were incubated with cell-permeant inhibitor of furin or plasmin, as indicated. αDG protein was purified from cell supernatant using anti-FLAG agarose and blotted using anti-FLAG or anti-αDG (IIH6) antibody. Cell lysates made from transfected C2C12 myotube cultures were also blotted with antibody to beta dystroglycan (βDG).

Next, we used molecular biology to eliminate each of the three potential furin cleavage sites in αDG, RSFR (75-79), RIRR (309-312), and RTPR (454-457). This was achieved by deleting the final R at the furin cleavage site to create single amino acid deletions delR79, delR312 and delR457. Two double mutants (delR79/delR312 and delR312/delR457) and a triple mutant (delR79/delR312/delR457) were also made. Each of these constructs, or wild type (WT) αDG, were transfected into CHO and CHO-LARGE cells. Recombinant proteins from the transfected cell supernatant were purified using anti-FLAG (M2) agarose and analyzed for protein expression and fragmentation by SDS-PAGE with immunoblotting for the N-terminal FLAG tag (Fig. 2). Transfection of wild type (WT) αDG predominantly produced cleaved N-terminal protein fragments, on average of about 40kDa, while little to no full-length protein was present. delR79 produced slightly more 120kDa protein than WT, but this mutant still was largely cleaved into a 40kDa fragment, while delR312 had minimal 40kDa fragment but high levels of 120-160kDa and 250 kDa protein. delR457, by contrast to del79 and del312, did not express much secreted protein at all. delR79/delR312 was mostly cleaved to 40kDa, while delR312/delR457 looked similar to the delR312. delR79/delR312/delR457 showed a pattern similar to delR312 but was more poorly expressed. Thus, the delR312 mutation was sufficient to inhibit the majority of N-terminal domain proteolysis on αDG.

**Deletion of asparagine 312 allows for secretion of full-length recombinant α dystroglycan (αDG) from CHO and CHO-LARGE cells.**
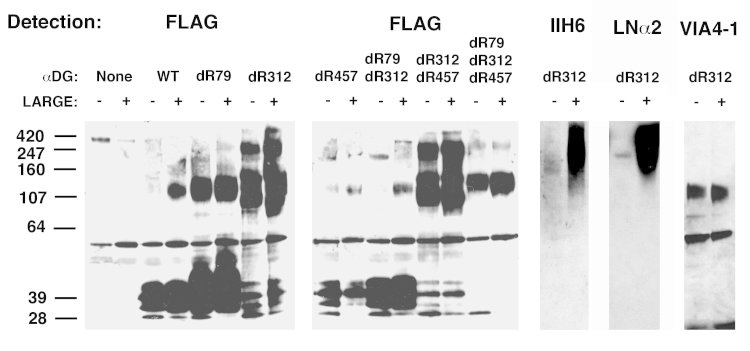
A plasmid encoding a full-length αDG cDNA (wild type, WT) with a FLAG epitope tag at the N-terminus, or various asparagine deletion mutants (dR), were transfected into CHO or CHO-LARGE cells and proteins purified from the supernatant using anti-FLAG agarose. Proteins were detected by immunoblotting for αDG using antibody to FLAG, αDG (IIH6 or VIA4-1) or by laminin α2 (LNα2) protein overlay.

For all seven αDG proteins studied, there was almost no apparent difference in the molecular weight profile by SDS-PAGE when comparing αDG purified from CHO and CHO-LARGE cell supernatant (Fig. 2). Despite this, binding of the IIH6 carbohydrate-dependent α dystroglycan antibody and binding of recombinant laminin α2 protein were dramatically increased on delR312 αDG purified from CHO-LARGE cells relative to CHO (Fig. 2). This was also the case for wild type protein and all other furin cleavage mutants (not shown). Interestingly, VIA4-1, another glycan-dependent antibody that specifically recognizes αDG, but that does not block laminin binding, as IIH6 does^29^, predominantly recognized the 120-160kDa glycoform of delR312 αDG, and did so equally well in protein purified from CHO-LARGE and CHO cells (Fig. 2). This was also the case for the del312/del457 double mutant (not shown). Thus, LARGE overexpression dramatically increased laminin binding, despite minimally changing the apparent molecular weight of delR312 αDG by SDS-PAGE. It is important to note that SDS-PAGE cannot really be used to assay total glycosylation in this context, particularly for highly glycosylated glycoproteins where gel migration profiles are not uniform.

To more explicitly demonstrate lack of cleavage at the R312 site in the delR312 mutant, we made and purified an anti-peptide αDG polyclonal antibody, DG2, to amino acids 298-312 (HIANKKPPLPKRIRR) in the dystroglycan protein coding sequence. This peptide contains the protein sequence just N-terminal to R312 furin cleavage site. We immunoblotted wild type (WT) and delR312 αDG protein purified from transfected CHO cell lysates and supernatants with this antibody (Fig. 3). While WT αDG secreted from CHO cells produced only 40kDa, cleaved, N-terminal fragment, delR312 protein produced only protein of only 160kDa or greater, again showing inhibition of proteolytic cleavage.

**Detection of uncleaved alpha dystroglycan using an antibody specific for the R312 furin cleavage site.**
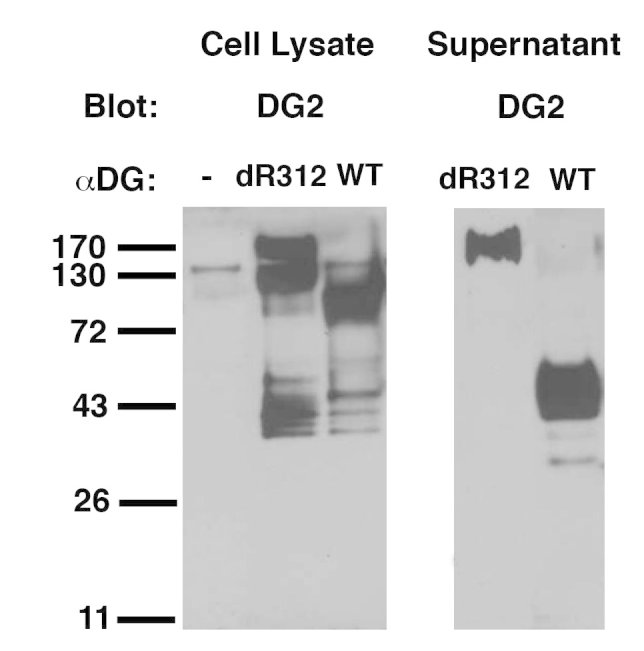
DG2, an affinity purified antiserum to the 15 peptides immediately N-terminal of the R312 protein cleavage site, was used to immunoblot cellular and secreted wild type (WT) and delR312 αDG protein after transfection of CHO cells.

We next developed a method to produce milligram amounts of purified FLAG-tagged delR312 αDG protein from transfected CHO and CHO-LARGE cell supernatant (Fig. 4). We used a continuous flow protocol to perfuse CHO or CHO-LARGE αDG(dR312)-transfected cell supernatant through packed M2 (anti-FLAG) agarose affinity columns. Once columns were loaded, they were washed and the FLAG-tagged protein periodically eluted with 2M MgCl_2_. We produced and purified αDG(dR312) protein from about 1.5L of cell culture supernatant by this method, doing two such preps each for delR312 αDG-transfected CHO and CHO-LARGE cells. After dialysis of eluted protein, we purified delR312 αDG further using Wheat germ agglutinin (WGA) agarose columns, followed by washing and elution with 0.3M N-acetyl-D-glucosamine (GlcNAc). Eluted protein (about 20mg from 1.5L) was extensively dialyzed to remove GlcNAc and salts prior to analysis.

**Method for purification of delR312 αDG secreted from transfected CHO cells.**
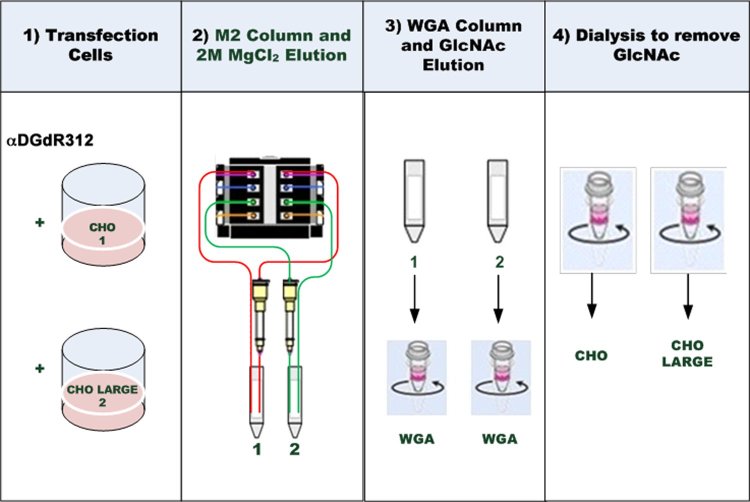
CHO cells or CHO cells stably overexpressing LARGE (CHO-LARGE) were transfected with a FLAG-tagged cDNA encoding delR312 αDG. Supernatant was continuously collected and applied to anti-FLAG antibody (M2) affinity resin, which was batch eluted in 2M MgCl2. After dialysis, protein was further purified over Wheat germ agglutinin (WGA) agarose and eluted with 0.3M N-acetyl-glucosamine (GlcNAc). After dialysis, delR312 αDG protein was concentrated and studied.

The identity of delR312 αDG was first confirmed using silver staining and Western blotting (Fig. 5). Analysis of 5 micrograms of post-purified protein showed silver staining of high molecular weight material at 160-250kDa consistent with native αDG in both CHO- and CHO-LARGE-purified material. In addition, CHO cell material had increased expression of several lower molecular weight bands, at 72kDa and 60kDa, relative to CHO-LARGE. The 72kDa co-migrated with a FLAG-positive band and the 60kDa band co-migrated with a band blotted by an antibody to the extreme C-terminus of αDG (ns). Thus, it is possible that these lower molecular weight bands represent cleaved fragments of αDG. Immunoblotting of post-purified material with antibody to FLAG showed protein bands from 160kDa to 250kDa for both CHO and CHO-LARGE material. Thus, as before, the glycoforms of αDG present in post-purified material appeared to be the same molecular weights in both CHO- and CHO-LARGE transfected cells. The molecular weight profile was roughly equivalent in profile between CHO and CHO-LARGE, though silver staining of gradient gel-separated material showed a slight apparent difference in the migration of high molecular weight (160-250kDa) material (Fig. 5).

**Characterization of delR312 αDG purified from transfected CHO or CHO-LARGE cells.**
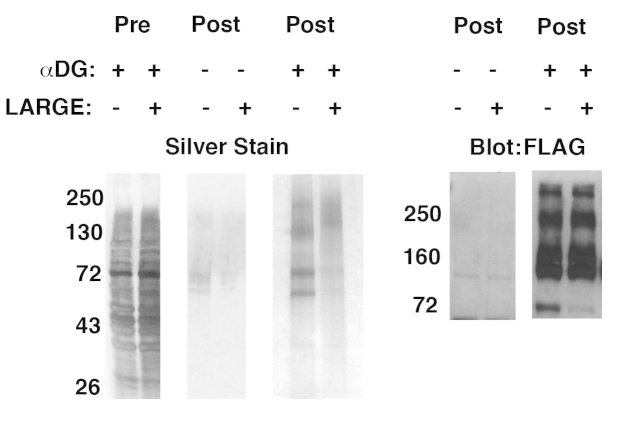
FLAG-tagged delR312 αDG was purified from the supernatant of transfected CHO or CHO-LARGE cells. 5μg of post-purified protein was compared to pre-purified supernatant and post-purified, but untransfected, supernatant by silver staining and anti-FLAG immunoblotting after separation by 4-12% gradient SDS-PAGE.

The identity of delR312 αDG from CHO and CHO-LARGE was also determined on 3 micrograms of sample by trypsin digestion followed by LC-MS/MS analysis. An example of one such sequenced peptide is shown in Fig. 6. Dystroglycan was the major protein identified by LC-MS/MS, with only bovine pancreatic trypsin inhibitor, which was added during purification, as a potential major contaminating protein. Bovine pancreatic trypsin inhibitor has no glycans that would interfere with subsequent αDG glycan analysis. Several other non-specific bands due to the preparation method, including trypsin and keratin, were also present (ns). Six common tryptic dystroglycan peptides were identified in CHO- and CHO-LARGE-purified delR312 αDG, at less than 5% false-detection rate (FDR), as follows: VTIPTDLIGSSGEVIK (aa80-95), LVPVVNNR (aa227-234), LGCSLNQNSVPDIR (aa262-275), KPPLPK (aa303-308), GGEPNQRPELK (aa490-500), and VDAWVGTYFEVK (aa506-517). The P values for identification of alpha dystroglycan in this material were 2.3x10^-9^ for CHO- and 1.2x10^-14^ for CHO-LARGE-produced protein. 4 unique peptides in N-terminal domain and 2 unique peptides in C-terminal domain were detected in all delR312 αDG preps. In addition, for delR312 αDG purified from CHO-LARGE cells, two more tryptic peptides were identified: VSTPKPATPSTDSSATTTRR (aa429-448) and LREQQLVGEK (aa540-549). Thus, sequencing of tryptic peptides from αDG purified from both CHO and CHO-LARGE cells by LC-MS/MS suggested the presence of full-length αDG protein.

**Mass spectra of a peptide from delR312 αG after purification from CHO-LARGE cells by continuous flow method.**
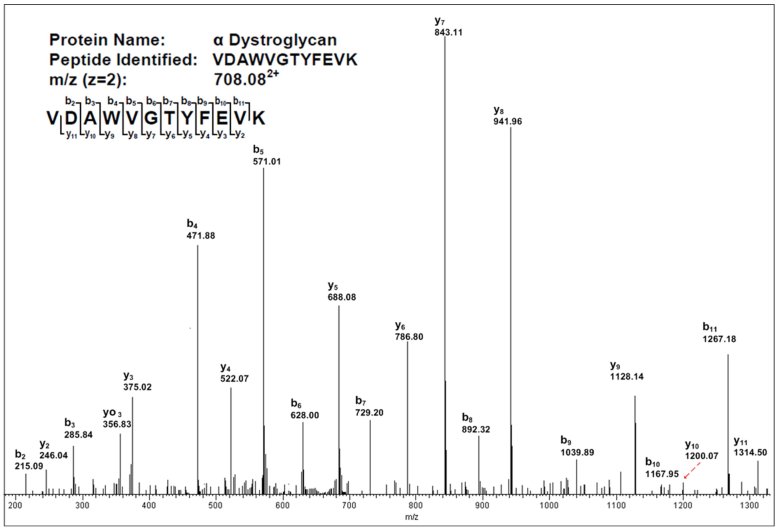
Peptide spectra determined by LC-MS/MS using shotgun proteomics.

The monosaccharide compositions of N- and O-linked glycans of αDG were analyzed by GC/MS as the TMS derivatives identified by their retention times and characteristic fragmentation patterns (Figs. 7 and 8, respectively). Glycans released by PNGaseF were validated by Western blotting with the M2 antibody (ns), confirming a 10-15kDa decrease in molecular weight, much as seen previously 29. Deglycosylated N-linked glycan samples were further analyzed for their O-linked glycan composition using beta-elimination of O-linked oligosaccharides. The sugars [Man, Gal, GlcNAc, GalNAc, NeuNAc] were identified by fragmentation pattern, ion traces from total ion chromatograms and selected ion monitoring at the respective masses (m/z 173: HexNAc, m/z 204: Hex, m/z 319: Hex-ol, m/z 378: HexNAc-ol). The total ion chromatogram (TIC) peak area data were used for quantitative determination of monosaccharides. Chromatograms of trimethylsilylated monosaccharides from N-linked and O-linked glycans of delR312 αDG purified from CHO and CHO-LARGE are shown in Figures 7 and 8, respectively. Trimethylsilylation of sugars released by hydrolysis gave multiple peak patterns by each sugar corresponding to tautomeric forms (α, and β-anomers, and pyranoside and furanoside rings). Therefore, in the spectra of the TMS ethers there is no single fragment ion that discriminates between the different monosaccharide classes, but these and their relative stereochemistries are readily discriminated by their retention times. Glycosaminoglycans and monosaccharides from decorin in equine systemic proteoglycan accumulation (ESPA) by GC/MS were successful after 2hr hydrolysis followed by acetylation with pyridine/acetic anhydride in methanol^30^, however, in this work, after the 16hr extended period of hydrolysis used to liberate glycan followed by acetylation, we were unable to detect glucuronic acid (GlcA). Thus, the use of a protocol did not allow for quantification of glycosaminoglycans.

**Chromatograms of monosaccharides from N- and O-linked glycans on delR312 αDG after purification from CHO cells.**
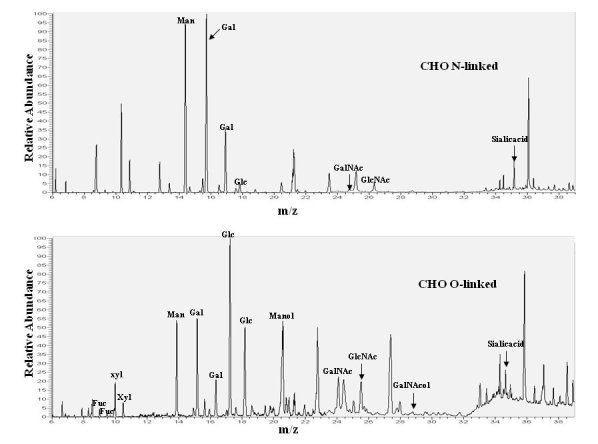
N-and O-linked monosaccharides were separated from del312 αDG purified from transfected CHO cells and analyzed by gas chromatography of their constituent monosaccharides as their trimethylsilyl derivatives, confirmed by standards, some of which are indicated.

**Chromatograms of monosaccharides from N- and O-linked glycans on delR312 aDG after purification from CHO-LARGE cells.**
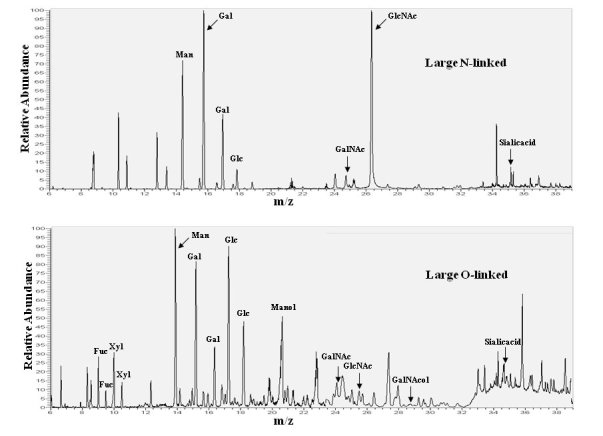
N-and O-linked monosaccharides were separated from delR312 aDG purified from transfected CHO-LARGE cells and analyzed by gas chromatography of their constituent monosaccharides as their trimethylsilyl derivatives, confirmed by standards, some of which are indicated.

Determination of the quantitative composition (mole %) of individual glyans on αDG was calculated from the experimental relative response data from monosaccharides referenced to known standard curves with inositol as an internal standard. Relative response curves for standard monosaccharide derivatives were uniformly linear, with R^2^ values as high as 0.99. Table 1 shows the monosaccharide composition of N-linked glycans of CHO and CHO-LARGE. Glucose was present as a contaminant in all chromatograms, and this probably originates from the chromatographic matrices used. The monosaccharide ratios of the N-glycans are based on GlcNAc as the core sugar are: Gal:Man:GlcNAc:NeuNAc =1.2:0.8:1.0:2.2 in CHO and =1.3:0.7:1.0:2.9 in CHO-LARGE. These four sugars in CHO and CHO-LARGE were consistently found to be the major constituents of N-linked glycans, at a molar ratio (CHO-LARGE/CHO) of 1.1:0.7:1.0:1.0. N-linked glycans in CHO-LARGE and CHO are likely to be the complex type, as roughly the same proportion of GlcNAc, Gal and Man was found in the samples. The total glycan content of N-linked sugars, relative to moles of protein, was similar between αDG isolated from CHO (4.4%) and CHO-LARGE (5.8%), suggesting that LARGE overexpression did not lead to extensive addition of sugars on N-linked structures.

**Table 1. Quantification of N-linked monosaccharides on αDG purified from CHO and CHO-LARGE cells.** Trimethylsilylated monosaccharides liberated from N-linked glycans were quantified by GC-MS using calibration curves prepared with standards. Errors are SD for n=2 experiments per condition.

	CHO	CHO-LARGE	CHO-LARGE/CHO
Monosaccharide	Mole%	Mole%	Mole% Ratio
Glc	1.7±0.7	2.9±0.0	1.7±0.4
Gal	19.5±1.6	21.5±2.4	0.9±0.1
Man	13.3±1.0	11.8±0.6	0.9±0.1
GlcNAc	17.7±0.5	16.2±1.4	0.9±0.1
NeuNAc	40.9±2.0	47.6±3.0	1.2±0.1

In the analysis of O-linked glycans, glucose was again present as a contaminant. Monosaccharide compositions from CHO and CHO-LARGE delR312 αDG were similar, with the presence of Gal, GalNAc, GlcNAc, GalNAc-ol, NeuNAc and Man-ol identified in both samples. GalNAc and Man were also detected as alditols, suggesting that the released glycans were originally O-linked to the protein backbone via GalNAc or Man residues at the reducing end. In addition, small amounts of fucose and xylose were detected (see Table 2). The relative amounts of monosaccharides in CHO-LARGE relative to CHO were constant for a number of monosaccharides (1.2:1.6:1.0:0.8:0.8) for Gal, Man, Man-ol, GlcNAc, GalNAc, respectively, somewhat different for GalNAc-ol and NeuNAc (5.2 and 0.4, respectively), and very different for fucose and xylose (14 and 43, respectively). Of these, xylose, fucose, and O-GalNAc were the only changes to reach statistical significance (P<0.05) using a standard two-tailed t test between CHO and CHO-LARGE. The increase in fucose and xylose for delR312 αDG from CHO-LARGE, relative to CHO, is based on their being only a very small amount of these monosaccharides in the CHO-purified material. Thus, even when increased in CHO-LARGE, fucose and xylose still only represented a small proportional fraction of the O-linked monosaccharides, about 5% each. Nevertheless, these data suggest a significant increase in fucose and xylose in the monosaccharide contents of O-linked monosaccharides on delR312 αDG purified from CHO-LARGE cells relative to CHO. The total O-glycan content in the two samples, however, was not significantly different. O-linked glycans were 33.3%, relative to protein, in delR312 αDG purified from CHO cells and 37.0% in delR312 αDG purified from CHO-LARGE cells. O-mannose linked glycans were almost exclusively detected on delR312 αDG from CHO and CHO-LARGE (Fig. 9). Mannitol, arising from O-linked mannose, represented 99±9% of O-linked sugar (as defined by GalNAcitol plus mannitol) on CHO cell delR312 αDG and 94±17% on CHO-LARGE delR312 αDG. Thus, delR312 αDG showed a very high concentration of O-man relative to O-GalNAc in both cell types.

**Table 2. Composition of O-linked monosaccharides on delR312 αDG purified from CHO and CHO-LARGE cells.** Trimethylsialylated monosaccharides liberated from O-linked glycans were quantified by GC/MS using calibration curves with standards. Errors are SD for n=2 experiments per condition.

	CHO	CHO-LARGE	CHO-LARGE/CHO
Monosaccharide	Mole%	Mole%	Mole% Ratio
Glc	19.8±0.8	16.6±0.4	0.8±0.1
Gal	6.1±0.6	7.6±0.7	1.2±0.2
Man	1.4±0.5	2.2±0.2	1.6±0.4
Man-ol	55.6±8.6	54.7±0.1	1.0±0.1
GlcNAc	2.6±0.2	2.1±0.1	0.8±0.1
GalNAc	0.9±0.1	0.7±0.2	0.8±0.1
GalNac-ol	0.6±0.1	3.1±0.3	5.2±0.7
NeuNAc	5.1±1.3	2.0±1.1	0.4±0.2
Fuc	0.5±0.1	6.9±0.3	13.8±1.7
Xyl	0.1±0.0	4.4±0.1	43±0.5

**Examples of chromatographic profiles and mass spectra for N- and O-linked monosaccharides from delR312 αDG purified from CHO and CHO-LARGE cells.**
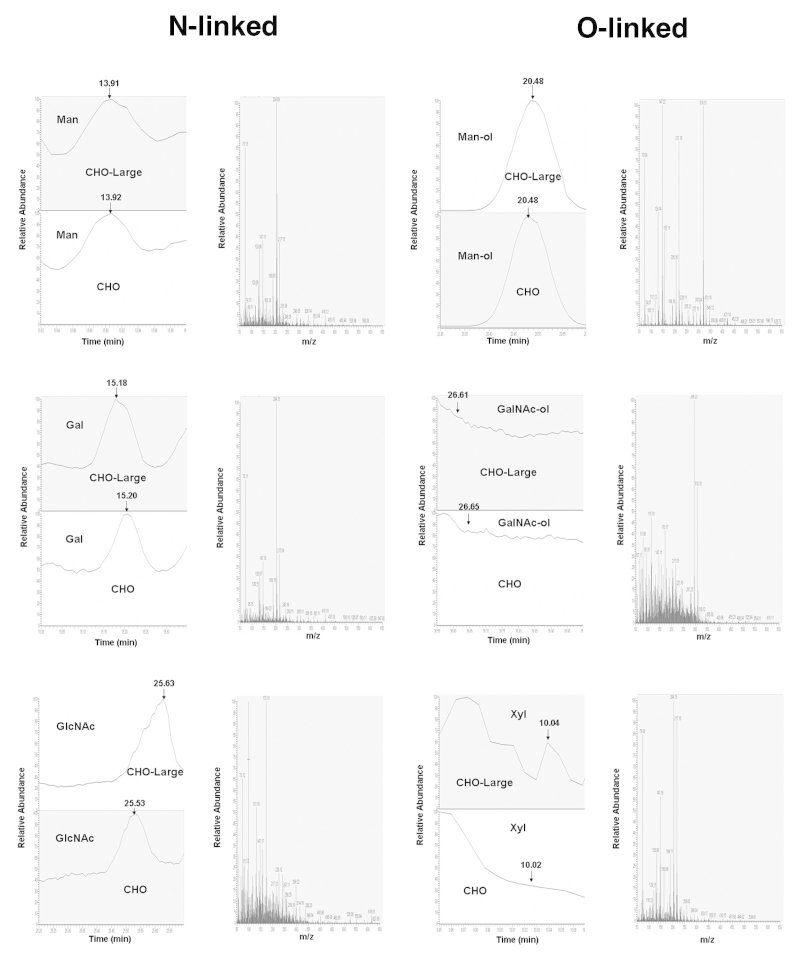
N- and O-linked monosaccharides liberated from delR312 αDG purified from CHO or CHO-LARGE cells and converted to their trimethylsilyl derivatives were separated by GC and analyzed by MS. N-linked monosaccharide examples are mannose (Man), galactose (Gal) and N-acetylglucosamine (GlcNAc). O-linked examples shown are mannitol (Man-ol), GalNAcitol (GalNAc-ol) and xylose (Xyl). Examples of single chromatographic profiles are shown from delR312 αDG purified from CHO and CHO-LARGE cells. Mass spectra shown are from CHO-LARGE samples.

## Discussion

It is now well known that genes responsible for the biosynthesis of the O-mannosyl-linked glycans on alpha dystroglycan (αDG) are required for proper binding of laminin and other extracellular matrix proteins to αDG and that defective glycosylation resulting from their mutation leads to various forms of dystroglycanopathy, which have skeletal muscle pathology and sometimes also pathology of the eye and brain^13^ ^31^. As αDG is a complex highly glycosylated glycoprotein, studies of its structure and function would be facilitated by methods that allow for production of large amounts of relatively pure glycoprotein. Here we have described one such approach to achieving this goal using transfected CHO cells. In addition, it is clear that in many dystroglycanopathies, overexpression of LARGE can lead to reintroduction of glycosylation motifs required for ECM binding, even in diseases where LARGE is the not the mutated gene^22^. LARGE stimulates the production of O-linked phosphor-mannose^20^ and is a tandem β1,3Xylα1,3GlcA glycosyltransferase^21^. LARGE2 also has the latter enzymatic activities^32^ ^33^. The current study does not discount any of this previous data and supports the notion that LARGE increases xylose content on αDG; αDG purified from CHO-LARGE cells showed a 43-fold increase in the molar content of xylose by GC/MS monosaccharide analysis. Unfortunately, the methods we utilized to release O-glycans did not allow for analysis of glucuronic acid (GlcA), the other glycan synthesized by LARGE. In addition, we made no effort to enrich for phosphorylated glycan fraction, which are enriched in LARGE-synthesized glycans.^21^

Despite its being cleaved off of the αDG protein in skeletal muscle, several studies have demonstrated the importance of the N-terminus of αDG in αDG function. Our use of a furin-resistant cleavage mutant allows for the study of full-length native αDG, and subsequent studies with various αDG deletion mutants should allow for a better understanding of the roles of various polypeptide fragments of αDG, both as glycosylation sites and as modifiers of glycosylation. Studies by Campbell and others have shown that the N-terminal region of αDG physically associates with LARGE and that such an association is required for LARGE overexpression to increase the expression of bioactive glycans recognized by the IIH6 monoclonal antibody 24. In addition, a missense mutation in the N-terminal region of αDG has been shown to give rise to a Limb Girdle muscular dystrophy, and this too correlates with reduced LARGE binding to the N-terminus as well as reduced IIH6 and laminin binding^25^. By deleting the furin cleavage site at asparagine 312, we have produced a protein that maintains the expression of this N-terminal domain as α dystroglycan migrates through the secretory pathway and becomes secreted from CHO cells. The one clear finding from utilizing this protein for our monosaccharide analysis is that the vast majority of the O-linked glycans on the delR312 αDG protein are O-linked mannose, with almost no O-linked GalNAc. This stands in contrast to studies on the furin-cleavable αDG studied by others. Studies by Wells^11^ and Hanisch^12^ both point to about two thirds of the O-linked glycan on αDG as being O-GalNAc, with O-mannose comprising the other third. Other studies similarly support the presence of a diversity of O-linkages^34^, with O-Man being in the minority^35^. That we get a different result with the furin-resistant αDG used here, both in CHO and CHO-LARGE cells, suggests that the maintained presence of the N-terminal domain to αDG may bias glycosylation towards O-mannosylation. Future studies will be required to verify that this is indeed the case. Other, far less interesting, alternatives that could explain this finding are that O-GalNAc activity on αDG is merely diminished in CHO cells or that N-acetyl groups on GalNAcitol were degraded during the protocol.

In addition to a 43-fold increase in the molar percentage xylose on O-linked monosaccharides for αDG(del312) purified from CHO-LARGE cells relative to CHO, we also found a 14-fold increase in the molar percentage of fucose. Dell and colleagues did find Lewis X (Galβ1,4(Fucα1,3)GlcNAc-R) on brain αDG, demonstrating that fucose can be present on this protein^9^. Xylose and fucose were both minor components of αDG purified from CHO cells, so their more dramatic increases could merely reflect the fact that these ratios were divided by a small denominator. Glucuronic acid was not appreciably detected in our experiments as done, so we cannot speak to that aspect of αDG glycosylation. These data demonstrate a method that should allow for biochemical analysis of αDG protein, or protein fragments, with respect to monosaccharide composition and other aspects of glycobiology.

## Materials and Methods

Creation of FLAG-tagged furin cleavage-resistant αDG mutants

In order to generate furin-resistant αDG, we used the following three oligonucleotides to individually eliminate the fourth R at each of the three potential furin cleavage sites, RSFR(76-79), RIRR(299-312), and RTPR(454-457)^5^:

delR79: 5’TTGGGCGCTCGTTTGTGACCATTCCAACAGATTTAATTGGC3’

delR312: 5’CCCAAGCGTATCCGACAGATCCATGCCACACCCAC 3’

delR457: 5’CCACGGACACCCCCGGTGCCACGGGTC 3’

Using QuickChange Multi Site Directed Mutagenesis kit (Stratagene, 200515, CA), we generated a total of six secreted forms of αDG mutants, all of which ended with a stop codon after the last amino acid of αDG and therefore lacked all of beta dystroglycan (βDG), as in 28. Mutants were made with N-terminal FLAG tag in pCMV1-FLAG vector as follows: αDG(delR79), αDG(delR312), αDG(delR457), αDG(delR79/R312), αDG(delR312/delR457) and αDG(delR79/delR312/delR457). All mutations were confirmed by DNA complete cDNA sequencing.

Cell Culture

Chinese Hamster Ovary (CHO) cells were obtained from American Type Culture Collection (ATCC). CHO cells were grown in DMEM with 10% fetal bovine serum (FBS) and 1% streptomycin and penicillin. CHO-LARGE cells were made by transfecting CHO cells with a myc-tagged cDNA encoding full-length LARGE. The plasmid containing the cDNA encoding LARGE was a gift from Pamela Stanley, Albert Einstein University^36^. This plasmid also contained a selectable IRES-neo^R^ cassette to allow selection of stable transfectants in G418. CHO-LARGE cells were selected and grown in 400 uG/mL G418, which led to cells that stably overexpressed LARGE protein, as previously described^36^. C2C12 cells, also originally from ATCC, were grown as myoblasts in DMEM with 20% FBS and 1% streptomycin and penicillin. To make C2C12 myotubes, C2C12 cells were grown to confluence and then fused for 3-6 days in DMEM with 2% horse serum and 1% streptomycin and penicillin, as previously described^37^. All cell lines were incubated at 37^o^C in 5% CO_2_-air mixtures in sterile, humidified, tissue culture incubators. Cell-permeant furin inhibitor (Dec-Arg-Val-Arg-Arg-CMK (chloromethyl ketone), Calbiochem 344930) and plasmin inhibitor (H-D-Val-Phe-Lys-CMK, Calbiochem 627624) were added to cells at 50uM for the full 2 days post-transfection following the manufacturer’s instructions.

Cell Transfection

C2C12 cells or CHO or CHO-LARGE cells were transfected with CMV1-FLAG-αDG or pCMV1-FLAG carrying different αDG mutants using Effectene transfection reagent (Qiagen) at 50-60% confluency. Transfected cells were cultured for 2-3 days prior to subsequent feeding. For transfected C2C12 cells, cells were co-selected in 400ug/mL G418 based after co-transfection with a 10-fold lower amount of a plasmid containing a neomycin resistance gene (neo^R^). Cells were then fused in low serum for 3-6 days to make myotube cultures, as previously described^37^.

Protein Purification

Media (10 ml x 60 plates) from transfected cells was cycled on a continuous basis through a packed M2 agarose columns as follows: Two columns, one each for untransfected or transfected CHO or CHO-LARGE cells, were packed with absorbent (Anti-Flag M2 agarose, 2 ml) and connected to channels of a peristaltic pump (Minipuls 3, Gilson). Each column was supplied with media (45 ml) from CHO or CHO-LARGE cells that had been transfected with an expression plasmid encoding full-length, furin-resistant, αDG (delR312 aDG) or media from mock-transfected control cells. Media were circulated through the columns at a flow rate of 0.15 ml/min overnight. The columns were washed with 45mL of buffer A (20mM Tris-Cl, pH 7.4, 100mM NaCl, 1:50 protease inhibitor cocktail (Roche, cOmplete Ultra tablets)) over 6 hours to remove unbound materials. The desired glycoproteins were eluted with 45 mL of Buffer B (20mM Tris-Cl, pH 7.4, 100mM NaCl, 2M MgCl_2_, 1:50 protease inhibitor cocktail) using a continuous flow system overnight. Dialysis, using 10,000MW cut-off dialysis tubing (Thermo Scientific, 68100), was carried out in Buffer C (20mM Tris-Cl, pH 7.4 with 1:50 protease inhibitor cocktail). Proteins were subsequently purified further in Buffer A using Wheat Germ Agglutinin (WGA)-coupled agarose columns, with elution in buffer A with 0.3M GlcNAc, as before^38^. Dialysis (10000MW snake-pleated, Thermo Scientific, 68100) was then repeated using buffer C.

SDS-PAGE, Silver staining and Immunoblotting

Proteins (2-10 μg total) were separated on 4-12% SDS-PAGE gels. Gels were processed for silver staining or immunoblotting. For silver staining, the gel was fixed overnight in 50% ethanol containing 5% acetic acid. On the following day, the gel was incubated in silver working solution (Silver stain kit, Thermo 24612) for 30 min and then further incubated in the reducing solution for 5 min. The gel was then washed in water and incubated in a stabilizer solution for 40 min. Alternatively, proteins separated by SDS-PAGE were transferred to nitrocellulose and analyzed by immunoblotting, as previously described^39^. Membranes were blocked in Tris-buffered saline (pH 7.4, with reduced (100mM) NaCl for IIH6 blots) with 0.02% Tween 20 (TBST) and 5% milk followed by incubating with IIH6 (Upstate Biotechnology, Lake Placid, NY) (1:2500) or anti-FLAG M2 monoclonal antibody (Sigma, 1:10000). Membranes were washed in TBST, incubated in anti-mouse IgM (IIH6), anti-mouse IgG (M2), or anti-Flag M2 conjugated to horseradish peroxidase, washed, and developed using the ECL chemiluminescence method.

Sequencing of purified of α dystroglycan by Nano-LC-MS/MS

The sample was lyophilized before being reduced in ammonium bicarbonate (50mM) containing DTT (10mM) at 55°C for 45 min, followed by carboxymethylation with iodoacetic acid (55 mM) at room temperature in the dark for 30 min. Reduced and carboxymethylated delR312 αDG was digested with sequencing grade-modified trypsin (1:30, Promega, Madison, USA) at 37°C overnight, followed by lyophilization.

All MS/MS experiments for peptide identification were performed using an LTQ-MS (Thermo Finnigan, USA) equipped with a nano-ESI source. The lyophilized peptide mixtures were solubilized in formic acid (0.1%) and loaded by an autosampler (Agilent 1100, USA) onto reversed phase columns (100 μm i.d. x 12 cm, Zorbax SB-C18 packing material). Solvent A consisted of H_2_O/CH_3_CN (95:5 v/v) + 0.1 % formic acid and solvent B of H_2_O/CH_3_CN (20:80 v/v) + 0.1 % formic acid. Samples (10mL) were eluted at ~200 nL/min (HPLC pump at 10 μL/min, split open). The RP-LC gradient consisted of: 0 to 10 % B (0-1 min), 10-45 % B (1-95 min), 45 to 60% B (95-110 min), 60 to 100 % B (110-115 min), 100 % B (115-120 min), 100 to 0 % B (120-121 min), and 100 % A (121-150 min).

Data-dependent MS acquisition conditions were as follows: 1 MS scan (3 microscans averaged) and 1 MS^2 ^on the top 13 most intense peaks; dynamic exclusion was enabled at repeat count 2, repeat duration 45s, exclusion list size 150, exclusion duration 45s, and exclusion mass width 1.5 m/z; collision-induced dissociation (CID) parameters were set at isolation width 3 m/z, normalized collision energy 35%, activation Q 0.25, activation time 30 ms. Both spectra were obtained at a heated capillary temperature of 200^o^C and an ESI voltage of 2.3 kV.

The fragment mass spectra were searched against a non-redundant database (NCBI) that included a contaminant database, using BioWorks (3.3.1SP1: ThermoFisher). The database search parameters included: only fully tryptic fragments were considered for peptide matching, the number of allowed missed cleavage sites was 2, the peptide tolerance was 2 Da, the fragment ion tolerance was 1 Da, and the capability to match one peptide sequence to multiple references within the database was set at 20. The calculations of FDR for our results were not obtained by applying the databases (original database and decoy database) due to insufficient data distribution. Instead, FDR for peptide matches in SEQUEST were estimated using the reverse sequence database strategy with transitional threshold at X_corr_ = 1.9 for z = 1, X_corr_ = 2.5 for z = 2, X_corr_ = 3.8 for z = 3^40^^-^ 42. IPI accession numbers for identification of proteins were entered from UniProt database (www.pir.uniprot.org).

Laminin overlays

The LG1-5 domains of the mouse laminin alpha2 were purified from transfected non-ionic detergent HEK293T cell lysates as previously described protocol^28^. Laminin α2 was used in blot overlays, again as previously described^43^.

Release of N-linked oligosaccharides

delR312 αDG, purified from CHO and CHO-LARGE cells (3mg each per experiment) and fetuin (1mg), as a control, were denatured in sodium phosphate buffer (50 mM, 200 µl, pH 7.5) containing SDS (0.5%) and DTT (0.04 mM) at 100^o^C for 10 min. The denatured samples were then diluted into non-ionic detergent-containing enzyme buffer treated with PNGase F (10 units from *Flavobacterium meningosepticum*, New England Biolabs, MA) at 37^o^C overnight following the manufacturer’s instructions. Digestions were terminated by heating the reaction mixture at 100^o^C for 3 min and enzyme removed by filter centrifugation (MW cut off 10 KD, Ultracel YM-10, Amicon). The centrifugates were lyophilized for the further analysis.

Release of O-linked oligosaccharides

Residues remaining on the filter following PNGase F digestion were centrifuged on the filter (MW cut off 50 KD, Ultracel YM-50, Amicon) to separate PNGase F from O-linked α-dystroglycan. Residues remaining on the filter were lyophilized. To identify the O-linked core sugars, the glycan chains were released from the protein by reductive β-elimination. O-linked α-dystroglycan from CHO or CHO-LARGE (3mg each) and fetuin as a control (1 mg), as well as inositol (5 μg) as an internal standard, were dissolved in NaOH (0.05 M)-NaBH_4_ (1M, 0.5 ml), vortexed and sonicated quickly. The samples were incubated for 16 hr at 45^o^C and then cooled to room temperature. After incubation, excessive NaBH_4_ was destroyed with 10 % acetic acid and samples were passed through column of AG 50W-X8 (H^+^) to remove Na^+^ ions, and boric acid was removed by repeated evaporations with methanol-acetic acid (9:1) at 45^o^C by N_2_.

Derivatisation of Monosccharides

Monosaccharide standards: xylose, fucose, glucose, galactose, mannose, N-acetylglucosamine, N-acetylgalactosamine, N-acetylmannosamine, glucosamine, arabitol, xylitol, fucitol, glucitol, galactitol, mannitol, N-acetylgalactosaminitol, glucuronic acid and galacturonic acid, fetuin and inositol, as an internal standard, were obtained from Sigma. Methanolic HCl (0.5M, 800 μl) was added to the samples and standards, which were kept for 16 hrs at 80^o^C. Samples and standards were dried down under N_2_ with anhydrous methanol (500 μl x 3). For trimethylsilylation, Tri-Sil (Pierce, 200 μl) was added and incubated for 30 min at 80^o^C. The mixture was concentrated to dryness with a stream of nitrogen and reconstituted in hexane (2 ml). The solution was filtered through a glass wool packed column and concentrated again. Samples reconstituted in hexane (1 μl) were analyzed by GC/MS. For acetylation, samples were added to anhydrous pyridine (50 μl) and acetic anhydride (50 μl) in methanol (200 μl) and incubated for 2 hrs at room temperature and then dried with N_2_.

Glycan composition analysis by Gas chromatography/Mass spectrometry (GC/MS)

GC/MS analyses were carried out using a gas chromatograph (Trace GC Ultra, Thermo Finnigan) equipped with a 30 m x 0.25 mm I.D. DB-1 column (Film 0.25mm, Agilent) connected to a mass spectrometer (Polaris Q, Thermo Finnigan). The split-splitless injector and transfer line temperature were set to 220^o^C and 270^o^C, respectively. The analytical conditions were as follows: Initial temperature 60^o^C, ramped to 260^o^C at 2^o^Cmin^-1^ and kept at 260^o^C for 3 min. The operating MS conditions were positive mode, scan rate 1scan s^-1^ over the range m/z 50- 650 and source temperature 200^o^C. EI (electron ionization) mass spectra were measured in the total ion-monitoring mode and peak area (TIC) data were used for quantitative determination via calibration curves using standards.

## Competing Interests

The authors have declared that no competing interests exist.

## Appendix 1

### Abbreviations

αDG, alpha dystroglycan; βDG, beta dystroglycan; CHO, chinese hamster ovary; DAG, dystrophin-associated glycoprotein; ECM, extracellular matrix; WT, wild type; TIC, total ion chromatogram
